# Effect of Hyperbaric Oxygen Therapy on whole blood cyanide concentrations in carbon monoxide intoxicated patients from fire accidents

**DOI:** 10.1186/1757-7241-18-32

**Published:** 2010-06-15

**Authors:** Pia Lawson-Smith, Erik C Jansen, Linda Hilsted, Ole Hyldegaard

**Affiliations:** 1 Laboratory of Hyperbaric Medicine, Department of Anesthesia, Center of Head and Orthopedics, Rigshospitalet, Blegdamsvej, Copenhagen, 2100, Denmark; 2 Center of Hyperbaric Medicine, Department of Anesthesia, Center of Head and Orthopedics, Rigshospitalet, Blegdamsvej, Copenhagen, 2100, Denmark; 3 Department of Clinical Biochemistry, Rigshospitalet, Rigshospitalet, Blegdamsvej, Copenhagen, 2100, Denmark

## Abstract

**Background:**

Hydrogen cyanide (HCN) and carbon monoxide (CO) may be important components of smoke from fire accidents. Accordingly, patients admitted to hospital from fire accidents may have been exposed to both HCN and CO. Cyanide (CN) intoxication results in cytotoxic hypoxia leading to organ dysfunction and possibly death. While several reports support the use of hyperbaric oxygen therapy (HBO) for the treatment of severe CO poisoning, limited data exist on the effect of HBO during CN poisoning. HBO increases the elimination rate of CO haemoglobin in proportion to the increased oxygen partial pressure and animal experiments have shown that in rats exposed to CN intoxication, HBO can increase the concentration of CN in whole blood.

**Objective:**

The purpose of the present study was to determine whole blood CN concentrations in fire victims before and after HBO treatment.

**Materials and methods:**

The patients included were those admitted to the hospital because of CO intoxication, either as fire victims with smoke inhalation injuries or from other exposures to CO. In thirty-seven of these patients we measured CN concentrations in blood samples, using a Conway/microdiffusion technique, before and after HBO. The blood samples consisted of the remaining 2 mL from the arterial blood gas analysis. CN concentration in blood from fire victims was compared to 12 patients from non-fire accidents but otherwise also exposed to CO intoxication.

**Results:**

The mean WB-CN concentration before patients received HBO did not differ significantly between the two groups of patients (p = 0.42). The difference between WB-CN before and after HBO did not differ significantly between the two groups of patients (p = 0.7). Lactate in plasma before and after did not differ significantly between the two groups of patients. Twelve of the 25 fire patients and one of the non-fire patients had been given a dose of hydroxycobalamin before HBO.

**Discussion and Conclusion:**

CN concentrations in blood from patients admitted to hospital with CO intoxication and smoke inhalation exposure did not differ significantly from controls. Accordingly, we were not able to detect any changes in CN concentrations in blood after treatment with HBO.

**Trial Registration:**

ClinicalTrials.gov identifier: NCT00280579

## Introduction

Reports have shown that patients admitted to hospital from fire accidents may have been exposed to cyanide (CN) gases as well as carbon monoxide (CO) [1-3]. Baud showed [4] that persons from fire accidents were both poisoned with CN and CO. CN is a potent intracellular poison which can be developed from incomplete combustion of materials containing nitrogen in fire accidents [5]. When fire temperatures reach 315°C (600°F) CN develops in the form of hydrogen cyanide (HCN) [1]. In the cell CN binds to the enzyme cytochrome oxidase a, a3 (CCO) (i.e. complex IV in the mitochondrial electron transport chain) similar to CO [6]; thus stopping the mitochondrial respiration chain and the formation of adenosine triphosphate (ATP).

According to several clinical studies, there is general agreement that HBO treatment is recommended in case of CO poisoning if the patient suffers from severe neurological symptoms or has been exposed to COHb concentrations higher than 25%. The current treatment indications for HBO therapy during CO poisoning in Denmark is in alignment with the recommendations as stated above [7-10]. Current treatment of CN poisoning is based on treating basic symptoms combined with hydroxycobalamin (Cyanokit^®^, OHCob) given i.v. [11,12]. OHCob i.v. reacts with any CN present in the blood stream and creates cyanocobalamin (B12 vitamin) a non-toxic substance that is excreted via the kidneys [13]. Whether OHCob can pass through the vascular wall and the blood-brain barrier to induce a direct detoxification effect within the central nervous system remains to be investigated [14]. However a French study by Astier et al demonstrated that OHCob may enter the intracellular compartment under in-vitro conditions [15].

HBO is recommended especially when supportive measures and other CN antidotes fail [16-18]. HBO is known to facilitate the dissociation of CO from cytochrome oxidase a, a3 in the mitochondrial respiratory chain [19].

In an animal model we have previously shown that HBO has an effect on the concentration of whole-blood cyanide (WB-CN) which increased significantly in comparison to untreated controls when measured 2 hours after HBO treatment [20]. Accordingly, the primary purpose of this clinical protocol was to measure whether HBO treatment in the CO and CN exposed patient would have the same effect on WB-CN in humans as demonstrated in the animal experiments. The secondary purpose of this clinical protocol was to determine how many patients from fires were CN poisoned above the toxic concentration levels defined as a whole-blood concentration higher than 39 μmol/L [11].

## Methods

The Local Ethics Committee approved the study. The study is registered at ClinicalTrials.gov identifier: NCT00280579. We studied 25 fire victims, aged 18 years or older, who were admitted to the level-1 trauma centre at Copenhagen University Hospital, Rigshospitalet for treatment of CO poisoning and smoke inhalation injury between January 2006 and November 2009. Twelve control subjects (i.e patients from non-fire accidents who were receiving HBO treatment for other causes such as suicide attempts or accidental gas inhalation) were also included.

Time sequence of blood sampling: Immediately before and after HBO therapy two 2 mL arterial blood samples were obtained from the patients. One sample was used immediately to determine the blood lactate concentration using the Radiometer ABL version 725 (Radiometer A/S, Copenhagen, Denmark). Any air bubbles were carefully removed from the other blood sample, and the syringe was tightly sealed and stored at -25°C until WB-CN concentration analysis the next day. Accordingly, WB-CN concentrations were determined before and after the first HBO treatment session. Two hours after the first acute HBO treatment session, a third blood sample for WB-CN concentration measurement was obtained. As controls we used blood samples from non-fire patients, collected and stored in the same way.

### Whole blood CN measurements

CN was measured using a Conway/microdiffusion method, where CN is liberated from blood in a gaseous phase and subsequently bound to OHCob forming cyanocobalamin [21]. The Conway chambers (Bel-Art products, Pequannock, NJ, USA) were placed on a heating plate and all reactions took place at 45°C. In the outer ring 1mL blood was mixed with 1 mL of 5% Triton X 100 and 2 mL of 50 μM OHCob in 0.067 M KH _2_PO _4 _was placed in the inner ring. Immediately before closing the chamber, 2 mL of 6.55 M sulphuric acid was added in the outer ring. In this acidic solution, the CN is in the protonated form, HCN, with a boiling point of 25.6°C. Thus any CN bound or free from the blood evaporates and binds to the OHCob in the inner ring and forms cyanocobalamin. Accordingly, previously given OHCob should not interfere with measurements of the cyanide concentrations in whole blood [21] After 30 min the solution in the absorbent chamber was aspirated and absorption at 361 nm was read (Shimadzu UV-1601 spectrophotometer, Shimadzu, Kyoto, Japan). The absorption increased linearly with the concentration of CN in the blood sample up to 100 μmol/L. All measurements were performed in duplicate and each series included a blank and three standards of 20, 40, and 100 μmol/L in 1 mol/L KOH. The lower limit of quantification was estimated to 20 μmol/L.

## Data analysis and statistics

Descriptive data are presented with mean and standard error of mean (SEM). Groups were compared with the Mann-Whitney test using SAS for Windows, version 9.1 (SAS Institute Inc., Cary, USA). P-values for CN concentrations are also presented with a quantification limit adjusted Mann-Whitney test, where all values below 20 μmol/L are analysed as 10 μmol/L.

## Results

### Effect of CN poisoning on WB-CN during HBO

#### General patient data

The patients' average age was 52 years. We included 37 patients of whom 19 were male and 18 female. Twenty-five patients were admitted from fire accidents and 12 patients were admitted to the Trauma Centre facility with CO poisoning from non-fire accidents. The average time from accident to arrival at the Trauma Centre was 3.22 hours. The time from arrival at the Trauma Centre to initiating HBO was 3.7 hours. Neither the time interval from accident to arrival, nor the time interval from arrival at the Trauma Centre to HBO treatment were initiated, showed any significant differences among patient groups.

#### Patients from fire accidents

Of the 25 patients from fire accidents, 2 had a WB-CN concentration higher than 39 μmol/L before HBO. OHCob was given to 12 patients, before HBO treatment, because of suspected cyanide poisoning.

In blood tests before and after HBO 13 showed an increase in WB-CN concentration and 10 a decrease. In the remaining two the WB-CN concentration did not change.

Of the 25 patients admitted from fire-accidents we were able to measure WB-CN concentration 2 hours after HBO therapy in 11 patients. Of these patients 2 had not received OHCob. No significant increase or decrease was observed in the 3 ^rd ^WB-CN measurement.

The mean WB-CN concentration on arrival for patients having received OHCob before the blood test was 15.4 μmol/L +/- SEM 4.1 μmol/L. In patients not receiving OHCob the mean was 14.33 μmol/L +/- SEM 1.4 μmol/L .The mean difference between CN concentration in blood before and after HBO in patients receiving OHCob was 2.1 μmol/L +/- SEM 1.4 μmol/L. Patients not receiving OHCob had a mean of 1.9 μmol/L +/- SEM 1.9 μmol/L.

The mean lactate concentration on arrival for patients receiving OHCob was 5 mmol/L and for patients not receiving OHCob 3.7 mmol/L. The mean lactate concentration after HBO was 1.9 mmol/L for patients receiving OHCob and 1.3 mmol/L for patients not receiving OHCob. See Table 1.

**Table 1:** Mean WB-CN, lactate and average COHgb

	Mean WB-CN at arrival	Mean WB-CN difference before and after HBO	Mean lactate before HBO	Mean lactate after HBO	Average COHgb before HBO	Average COHgb after HBO
**Patients from fires receiving OHCob** **N = 12**	15.4 μmol/L	2.1 μmol/L	5 mmol/L	1.9 mmol/L	27.9%	2%

**Patients from fires not receiving OHCob** **N = 13**	14.33 μmol/L	1.9 μmol/L	3.7 mmol/L	1.3 mmol/L	23%	1.9%

**Patients from non-fires** **N = 12**	12.5 μmol/L	1.1 μmol/L	4.4 mmol/L	2.5 mmol/L	25.9%	2.9%

Mean WB-CN, lactate and carbon monoxide concentrations before and after HBO therapy in all three groups. N = number of patients.

#### Patients from non-fire accidents

None of the 12 patients from non-fire accidents had a CN concentration in whole blood higher than 39 μmol/L before HBO. OHCob was given to one patient before leaving the level-1 Trauma Centre thus before HBO. In blood tests before and after HBO seven patients showed an increase in WB-CN and five a decrease. Of the 12 patients from non-fire accidents we were able to measure WB-CN 2 hours after HBO therapy in 3 patients, of these which only 1 had received OHCob. No significant increase or decrease was observed in the 3 ^rd ^WB-CN measurement.

The mean WB-CN concentration on arrival was 12.5 μmol/L +/- SEM 1.5 μmol/L. The mean difference between CN concentration in whole blood before and after HBO was 1.1 μmol/L +/- SEM 0.6 μmol/L.

The mean lactate concentration on arrival was 4.4 mmol/L. The mean lactate concentration after HBO was 2.5 mmol/L. See Table 1.

#### Patients from fire accidents compared with patients from non-fire accidents

The mean WB-CN concentration on arrival did not differ significantly between the three groups of patients. See Table 2. WB-CN concentrations before and after HBO differ significantly in the three groups of patients. See Table 2.

**Table 2:** Comparison of WB-CN

	**Patients from fires receiving OHCob**		Patients from fires not receiving OHCob	
	N = 12		N = 13	
	**WB-CN at arrival**	**Differences in WB-CN before and after HBO**	**WB-CN at arrival**	**Differences in WB-CN before and after HBO**

**Patients from fires not receiving OHCob**	P = 0.72	P = 0.07		
N = 13				

**Patients from non-fires**	P = 0.4	P = 0.16	P = 0.48	P = 0.7
N = 12				

When comparing WB-CN concentration on arrival at the Trauma Centre or WB-CN concentration before and after HBO amongst all three groups there are no significant differences. P = p-values. N = number of patients.

Lactate concentrations in plasma before and after HBO treatments did not differ significantly in the three groups of patients.

## Case stories

We found two CN poisoned patients with WB-CN concentrations higher than 39 μmol/L, and include the case stories to illustrate the course of treatment following fire accidents with suspected of CO and CN poisoning.

### Case 1

In 2007 a fire victim patient was transported from primary hospital to our level 1 Trauma Centre. The patient was rescued from a burning apartment where the patient was lying in a cloud of smoke up to1,5 meters. Arterial puncture at a primary hospital showed a carboxyhaemoglobin (COHb) of 40% and lactate of 8,2 mmol/L in combination with alcohol intoxication. During ambulance transport, the patient shortly regained consciousness, but was unconscious again on arrival at primary hospital. Due to possible inhalation injuries of the upper airways, the patient was sedated with propofol and fentanyl and intubated at primary hospital before transportation to Rigshospitalet for subsequent HBO treatment. Blood tests for CN were taken on the arrival to Rigshospitalet including a new arterial puncture. This showed pH 7.33, pCO _2 _6.6, pO _2 _6.65 , BE 0.2, COHb of 17% and lactate of 4.2 mmol/L. Subsequently, the patient was given OHCob as CN antidote. The WB-CN concentration before antidote treatment was 58 μmol/l. There were plenty of soot particles in the tube; accordingly, bronco alveolar lavage (BAL) was considered, but it was decided that HBO had first priority. The patient received 3 HBO therapies during the first 24 hours. After the first 2 HBO treatments BAL was performed and showed intact mucous membranes coated with a large amount of soot particles. COHb normalised after the first HBO therapy to 2.8% and WB-CN concentration was 28 μmol/l. After 24 hours the CN level in blood was still high (23 μmol/l) in spite of OHCob. During the hospitalisation the patient received 5 HBO treatments. The patient was extubated 5 days later without problems and transferred to the neurological unit.

### Case 2

An unconscious patient was rescued from a burning apartment with a Glasgow Coma Scale 3. The patient was intubated and brought to the level 1 Trauma Centre. Blood tests for CN were taken on the arrival including an arterial puncture for blood gas analysis. The arterial blood sample showed a COHb of 33.5% and lactate of 9.0 mmol/L. After the blood tests the patient received OHCob. The patient had 2 ^nd ^and 3 ^rd ^degree burns on 17% of the body, arms and face. A BAL performed in the Trauma Centre showed soot particles coating of the mucous membranes in the trachea and major bronchi. The patient was sent to the hyperbaric chamber for HBO treatment. Immediately before HBO a blood sample was taken that showed pH 7.32, pCO _2 _5.65, pO _2 _23.5 , BE -3.7, COHb of 10.2% and plasma-lactate of 4 mmol/L. The patient received 2 HBO treatments before being admitted to the Intensive care unit. After the first HBO treatment the arterial puncture showed pH 7.3, pCO _2 _6.23, pO _2 _11.8 , BE -3.6, COHb of 3.5% and plasma-lactate of 2.8 mmol/L. The WB-CN concentration was 39 μmol/l before and 14 μmol/l after the first HBO treatment. Two hours after HBO the WB-CN concentration was 13 μmol/l. During the next 5 days the patient developed septic shock followed by multiple organ dysfunction syndrome caused by the severe burn injuries. On the 5 ^th ^day the patient developed hyperthermia, which was complicated by cardiovascular collapse and a fatal irreversible circulatory arrest.

## Discussion

In CN poisoning the toxic concentration in blood has been reported to be 39 μmol/L and higher than 100 μmol/L is potentially lethal [11]. In the group of patients from fire accidents only 2 patients had a WB-CN concentration higher than 39 μmol/L and 1 of those received OHCob before the blood test. In the group of non-fire patients none had a CN concentration higher than 39 μmol/L and only 1 patient received OHCob. Often patients were transferred to the level-1 Trauma Centre from other hospitals before HBO and consequently, the WB-CN blood tests were taken hours after the patients had been exposed to CN. Following absorption CN is rapidly distributed throughout the body. Because of the time delay from possible CN exposure until blood sampling, most of the CN would have distributed to the tissues before the blood sample was taken. Therefore, it cannot be excluded that some of the patients who were exposed to fire have had higher concentrations of CN in their blood, than we were able to measure. We therefore recommend, if possible, taking the WB-CN test at the scene of the emergency.

In an earlier study at Rigshospitalet the result was similar, showing that only 2 of 40 patients had a CN concentration higher than 39 μmol/L even though a greater number of patients were expected to have CN poisoning [22]. The study by Meyhoff et al. [22], included two control groups of which one was consisting of smokers and the other non-smokers. Their results showed that WB-CN concentrations were marginally higher in smokers compared with non-smokers and that there were no statistical differences between these groups. Accordingly, there does not seem to be any effect of smoking on CN levels on whole-blood measurements. Based on these observations, the current study did not evaluate or register whether or not patients admitted with severe CO-poisoning were smokers.

The Conway method does not show how much CN is stored in the tissues. It has been demonstrated, that once CN is absorbed, the body will distribute it with 50% present in blood, 25% in muscle and 25% in other of the organs, predominantly in the liver and brain [23]. This also applies to other available methods currently used for measuring CN concentrations. There is a major need for a new method of measuring CN. Because of rapid redistribution, the measured WB-CN concentration is likely to be too low.

Before HBO the WB-CN concentration in fire victims did not differ significantly from patients not exposed to fire or smoke inhalation. One explanation may be that the fire victims in the present study were not CN poisoned. This does not corresponds with earlier studies [1-4]. Another possible explanation for this observation may be found in the delay from the time of CN exposure and HBO treatment. The CN half-life in whole blood is only 1 hour [21]. Consequently, CN may be irreversibly stored in the cells by the time the patients receive HBO. This may seem at variance with the findings of Lawson-Smith et al. [20], where WB-CN concentrations measured in rats exposed to CN poisoning, were found to increase after HBO therapy [20]. However, in the Lawson-Smith et al. study [20], rats were exposed to significantly higher doses of cyanide and received HBO closer to the CN exposure as compared to the patients in this report. We were not able to detect any differences in WB-CN concentrations before and after HBO. Nor did the WB-CN concentrations differ significantly in the two groups. This does not mean that HBO treatment will not be of benefit for the patient suffering from tissue hypoxia caused by CN poisoning, but rather that the WB-CN concentration in our patients were too low to detect any differences before and after HBO therapy. In our relatively small sample, we were not able to produce a correlation between WB-CN concentrations and plasma lactate concentrations. (See Table 1) as previously demonstrated by Baud et al. [24]. Nevertheless HBO did cause a substantial reduction in plasma lactate and COHb (See Table 1).

Clinicians are often unable to diagnose cyanide poisoning in the emergency setting [25] and it is often difficult to find the patients with a WB-CN concentration higher than 39 μmol/L [11]. Clinicians often treat patients with OHCob because of the history of fire accident. As mentioned above, OHCob converts CN to a non-toxic substance being cyanocobalamin (B12-vitamin). Cyanocobalamin is excreted through the kidneys [13] and has a safe side effect profile making it safe to infuse even without a clear diagnose of CN poisoning. Side effects are red colouring of skin and urine, urticarial eczema and seldom anaphylactic chock is seen [14]. Williams et al found that OHCob accelerates the renal excretion of CN when CN was administered as NaCN [26]. Whether this is the case following HCN intoxication from fire accidents remains unknown.

In patients hospitalized with a history of fire accident, combined with severe neurological symptoms such as reduced Glasgow Coma Scale Scoring and either soot particles in the mouth or tracheal expectoration, is likely to be indicative of CN poisoning [11]. In this report, a majority of patients from fire accidents received OHCob as apposed to 1 patient from non-fire accidents. In keeping with the apparently safe side effect profile of OHCob and the damage CN poisoning may cause if left untreated, we recommend the use of OHCob infusion even if the WB-CN concentration has not been confirmed by direct measurement. Reports have shown that survivors of CN poisoning may have permanent sequelae in form of brain damage. CN is recognized as a cause of permanent neurological disability, ranging from various extrapyramidal syndromes to post-anoxic vegetative states [27]. This has been shown by high-resolution magnetic resonance imaging and positron emission tomography [28]. The area is not well investigated and will require more studies in the future.

Of the 25 patients admitted to the hospital from fire accidents, at least 2 patients were exposed to toxic levels of CN. In view of the limitations with respect to the time delay from CN intoxication and WB-CN concentration measurements, we conclude that patients exposed to fire accidents may well suffer from CN intoxication. Accordingly, they should be treated with a suitable antidote even in the absence of verified blood CN measurements and preferably in combination with HBO treatment, the latter being the primary treatment if combined with CO poisoning.

## Competing interests

The authors declare that they have no competing interests.

## Authors' contributions

PL-S participated in the design and coordination of the study, performed the statistical analysis and drafted the manuscript. LH participated with blood sample analysis and whole blood cyanide assays. ECJ conceived of the study and participated in the design. OH conceived of the study, participated in the sequence alignment as well as the design and coordination of the study. All authors read and approved the final manuscript.
